# Effectiveness of Telemetry Guidelines in Predicting Clinically Significant Arrhythmias in Hospitalized Patients

**DOI:** 10.4021/cr129w

**Published:** 2012-01-20

**Authors:** Sandeep K. Dhillon, JosephTawil, Baruch Goldstein, Dayana Eslava-Manchego, Jagdeep Singh, Sam Hanon, Paul Schweitzer, Steven R. Bergmann

**Affiliations:** a Division of Cardiology, Department of Internal Medicine, University Hospital and Manhattan Campus for the Albert Einstein College of Medicine-Beth Israel Medical Center, New York, USA

**Keywords:** Telemetry, Arrhythmias, Clinically significant arrhythmias, Guidelines, Monitoring

## Abstract

**Background:**

Cardiac rhythm monitoring is widely applied on hospitalized patients. However, its value has not been evaluated systematically.

**Methods:**

This study considered the utility of our institutional telemetry guidelines in predicting clinically significant arrhythmias. A retrospective analysis was performed of 562 patients admitted to the telemetry unit. A total of 1932 monitoring days were evaluated. Patients were divided into 2 groups based on telemetry guidelines: “telemetry indicated” and “telemetry not indicated”.

**Results:**

Differences in arrhythmia event rates and pre-defined clinical significance were determined. One hundred and forty-four (34%) vs. 16 (11%) patients had at least one arrhythmic event in the “telemetry indicated” group compared with the “telemetry not indicated” group, respectively (P = 0.001). No patient in the “telemetry not indicated” group had a clinically significant arrhythmia. In contrast, of patients in the “telemetry indicated” group who had at least one arrhythmic event, 36% were considered clinically significant (P < 0.05).

**Conclusion:**

In conclusion, this study validates and supports the use of our institutional telemetry guidelines to allocate this resource appropriately and predict clinically significant arrhythmias.

## Introduction

Cardiac rhythm monitoring, initially employed in Coronary Care Units during the 1960s to decrease arrhythmic death after myocardial infarction, is now broadly applied in a wide variety of clinical settings. Telemetry monitoring is a valuable resource for patients hospitalized with a range of cardiac and non-cardiac diseases. However, although many hospitalized patients are monitored for arrhythmias, the actual incidence of clinically significant arrhythmias is low [1]. Further, many arrhythmias noted incidentally on telemetry are of little or no clinical importance. Additionally, telemetry artifact may be mistaken for a true arrhythmia, leading to unnecessary tests or treatments.

Few studies have defined the value and limitations of telemetry in a systematic fashion. In addition there are few published criteria for inpatient telemetry [2]. Our hospital developed and implemented guidelines to identify patients who require telemetry (Appendix A, www.cardiologyres.org). The purpose of the present study was to assess the incidence and clinical significance of arrhythmias in patients on telemetry and to establish the effectiveness of the guidelines in predicting these events in order to validate our institutions telemetry guidelines.

## Methods

A retrospective analysis was performed of all patients admitted to the inpatient telemetry unit of the Beth Israel Medical Center over the course of six consecutive months. Patients admitted to an Intensive Care Unit were excluded. The study was approved by the Institutional Review Board of the Beth Israel Medical Center.

The telemetry units employ Philips Intelvue monitors (Philips Health Care Bothell, WA). These have pre-set automatic triggers. In addition, telemetry was monitored continuously (24 hours/day) by trained technicians who manually note and document arrhythmias. Institutional telemetry guidelines were developed and implemented (Appendix A, www.cardiologyres.org) [3]. They are disseminated and reinforced semi-annually. However, the decision for telemetry admission is at the discretion of the individual treating physician(s).

The medical record including all available telemetry data were reviewed and analyzed. Demographic information, medical history and the indication for admission was collected. Patients were divided into 2 groups based on their fulfillment of the telemetry guidelines: “telemetry indicated” and “telemetry not indicated”.

A total of 562 telemetry admissions, encompassing 1,932 days of monitoring were included. The indication for telemetry was recorded for each day the patient was monitored. The stored data was reviewed by 2 authors for the occurrence of arrhythmias, including ventricular fibrillation, sustained ventricular tachycardia (lasting > 30 seconds), nonsustained ventricular tachycardia (> 3 beats and lasting < 30 seconds), idioventricular rhythm, supraventricular tachycardia, atrial fibrillation or atrial flutter with rapid ventricular response (HR >110 bpm), sinus bradycardia (heart rate < 50 beats per minute), pauses > 3 seconds, junctional rhythm, or second and third degree atrioventricular block. Single atrial and ventricular premature beats were not recorded as arrhythmic events.

In addition to analyzing the incidence of arrhythmias, the clinical significance of these arrhythmias was also determined from pre-defined criteria. Arrhythmias were defined as clinically significant if they triggered a change in management, including a medication change (not including the repletion of electrolytes), cardioversion, electrophysiology study, or transfer to an Intensive Care Unit. Telemetry was reviewed by 2 authors experienced in cardiac rhythm analysis who were blinded to the clinical information. Differences of opinion were adjudicated by a 3rd author. The clinical and demographic data was reviewed by an author blinded to the telemetry data.

### Data analysis

Demographic data were analyzed using Chi-square test when comparing proportional data. The Fisher exact test was used if the subsets were too small for a Chi-square test. The Wilcoxon test was used to compare the two groups on continuous measures. The Jonckheere-Terpstra test was used to compare the two groups on ordinal outcomes. A P < 0.05 was considered statistically significant.

## Results

**Figure 1:**
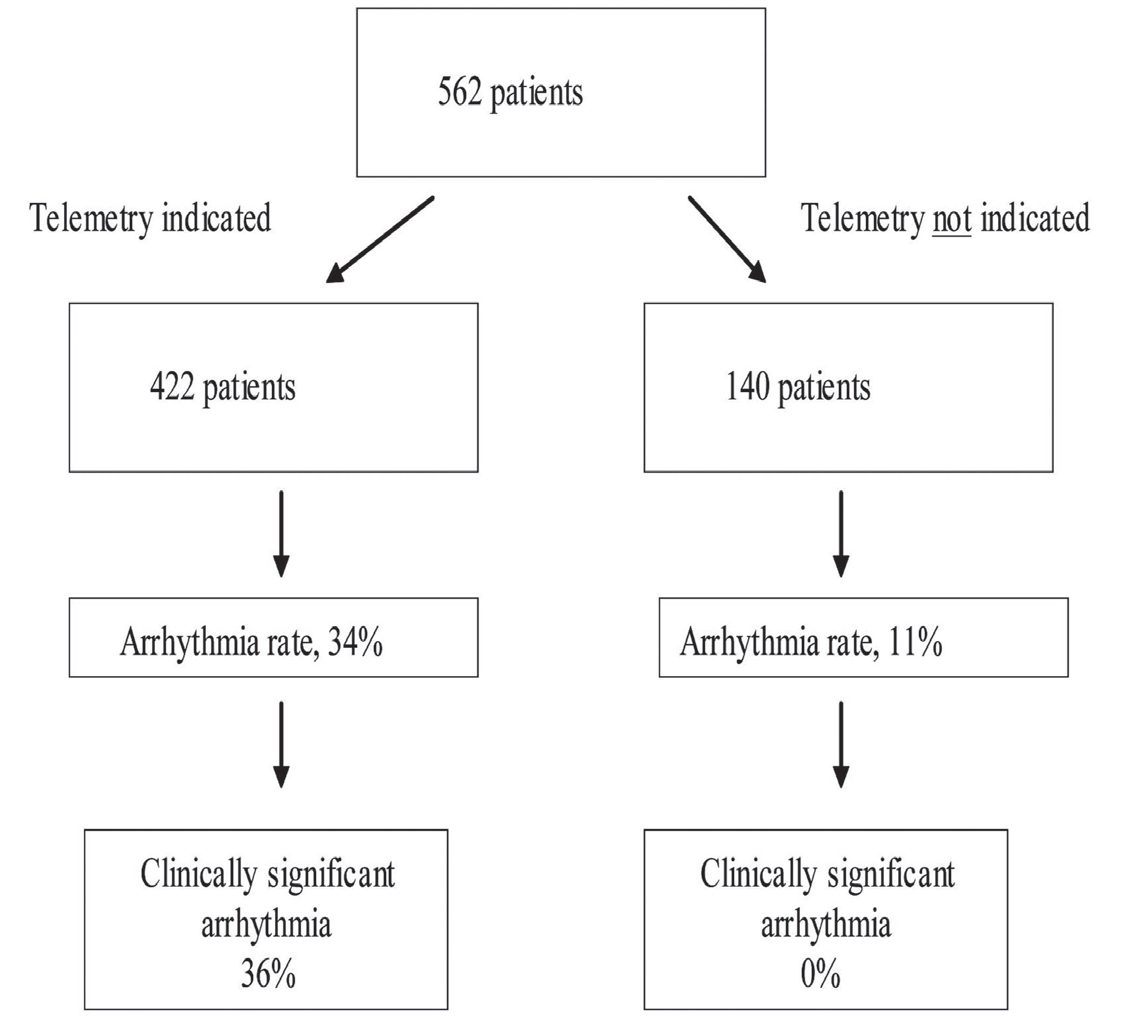
Total telemetry admissions and their distribution.

Of the 562 patients admitted to telemetry, 422 (75%) met telemetry monitoring criteria (“telemetry indicated” group) and 140 (25%) did not meet our institutional criteria for telemetry monitoring (“telemetry not indicated” group) (Fig. 1). The baseline characteristics of the two groups are shown in Table 1.

**Table 1:** Patient Demographics and Baseline Clinical Characteristics

		Telemetry indicated	Telemetry not indicated	P value
Age (years)	Mean (SD)	66 (15)	64 (17)	n.s
Gender	Male %	54%	36%	0.008
Ethnicity	Asian/Pacific Islanders	5%	10%	n.s.
	Black	20%	13%	n.s.
	Non-black Hispanic	33%	24%	n.s.
	White	41%	51%	n.s.
	Other	< 1%	1%	n.s.
Past Medical History	Diabetes Mellitus	36%	27%	n.s.
	Hypertension	78%	66%	0.045
	Dyslipidemia	47%	30%	0.013
	Prior MI	22%	6%	0.002
	Heart Failure	25%	10%	0.007
	Arrhythmia	26%	10%	0.006
	Atrial Fibrillation	15%	10%	n.s.
	PPM/AICD	9%	6%	n.s.
	CAD	40%	17%	0.0004
	Smoker			
	Never	49%	63%	n.s.
	Ever	31%	20%	n.s.
	Current	20%	17%	n.s.
Past Surgical History	PCI	20%	10%	0.049
	CABG	3%	6%	n.s.
	Other cardiac surgery	3%	3%	n.s.

MI: Myocardial Infarction; PPM: Permanent pacemaker; AICD: Automated implantable cardioverter defibrillator; CABG: Coronary artery bypass graft; PCI: Percutaneous coronary intervention.

There were significantly more men in the “telemetry indicated” group compared with the “telemetry not indicated” group (54% vs. 36%; P < 0.008). In addition, the “telemetry indicated” group had a significantly higher prevalence of hypertension, dyslipidemia, prior myocardial infarction, congestive heart failure, history of arrhythmias, coronary artery disease, and prior coronary interventions (Table 1).

The clinical indications for the telemetry monitoring for the 422 admissions to the “telemetry indicated” group are shown in (Table 2). Indications were divided into rule out acute coronary syndrome (ACS); ACS; assessment and control of significant arrhythmias; syncope that is suspected to be cardiac in origin; and others (Appendix A, www.cardiologyres.org). Most patients had one primary indication for the telemetry monitoring but 110 (26%) of the patients had more than one indication for telemetry monitoring.

**Table 2:** Clinical Indication for Telemetry Monitoring in the “Telemetry Indicated Group”

Indications for telemetry		% of patients
Rule out ACS	R/O ACS alone	42.6
	R/O ACS + Arrhythmias	9.9
	R/O ACS + Syncope	6.1
	R/O ACS + Others	4.2
ACS	ACS alone	9.4
	ACS + Arrhythmias	2.3
	ACS + Syncope	0
	ACS + Others	0
Arrhythmias	Arrhythmias alone	5.2
	Arrhythmias + Syncope	0.9
	Arrhythmias + Other	0.47
Syncope	Syncope alone	5.2
	Syncope + Others	0.47
		
Others	Others	10.9
	Others ± R/O ACS ± Arrhythmias ± Syncope	1.8

ACS: Acute coronary syndrome.

**Figure 2:**
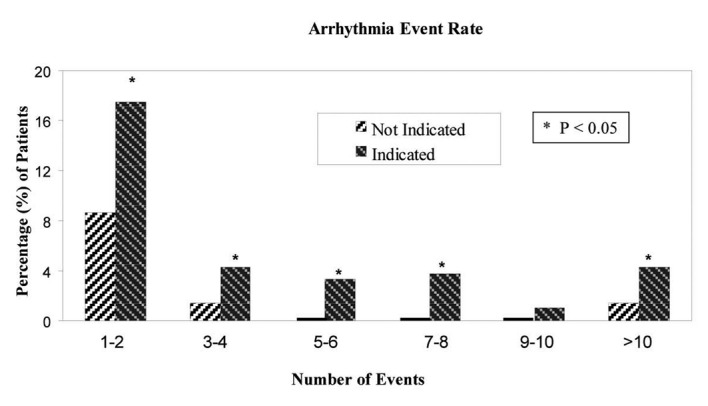
Comparison of the arrhythmia event rate in the “telemetry not indicated” verses “telemetry indicated group” for patients with atleast one arrhythmic event. Arrhythmia events were more frequent in patients who were in the “telemetry indicated” group.

In the “telemetry indicated” group, 144/422 patients (34%) had total of 336 arrhythmic events. In contrast, in the “telemetry not indicated” group, only 16/140 patients (11%) had total of 53 events (P < 0.001). Figure 2 shows the percentage of patients with events on telemetry in both groups with respect to the day of monitoring. The qualifying arrhythmic events identified are listed in Table 3.

**Table 3:** Electrocardiographic Events Identified by Telemetry

Arrhythmia identified	Indicated	Not indicated	P value
Ventricular Fibrillation	0	0	n.s
Sustained Ventricular Tachycardia (> 30 seconds)	0	0	n.s
Non-sustained Ventricular Tachycardia (> 3 beats but less 30 seconds)	93	15	n.s.
Supraventicular Tachycardia (HR > 110, non-sinus)	21	8	0.042
Atrial Fibrillation with rapid ventricular response (HR > 110 bpm)	80	4	0.0075
Atrial Flutter with rapid ventricular response (HR > 110 bpm)	9	3	n.s
Sinus Bradycardia (HR < 50 bpm)	110	22	n.s
Pause > 3 seconds	5	1	n.s
Junctional Rhythm	0	0	n.s
Third-degree type II atrioventricular block	1	0	n.s.
Second-degree type II atrioventricular block	0	0	n.s.
Second-degree type I atrioventricular block	0	0	n.s.
Idioventricular Rhythm	17	0	n.s.
Total Number of Events	336	53	0.001

In those patients having at least one arrhythmia, there were 1.26 ± 1.94 arrhythmias per 24 hours in the first 48 hours of monitoring in the “telemetry indicated” group, compared with 0.56 ± 0.50 arrhythmias per 24 hours in the first 48 hours in the “telemetry not indicated” group (P = 0.16).

**Figure 3:**
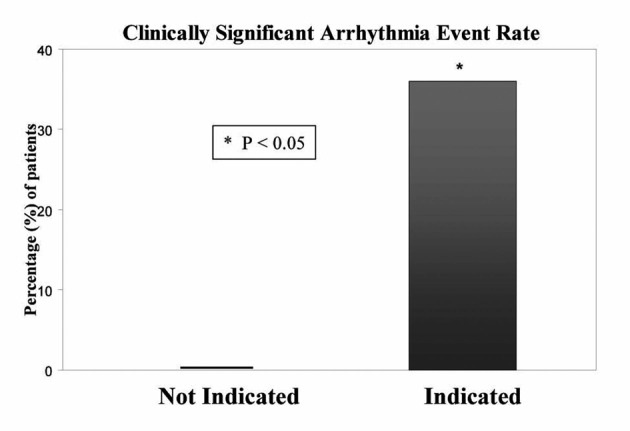
Comparison of clinically significant event rate in indicated verses the not indicated group. Of the patients with at least one event on telemetry, 36% of the patients in the indicated group had clinically significant event verses 0% in the not indicated group.

Among the 144 patients in the “telemetry indicated” group who had at least one arrhythmic event, 36% of the arrhythmias were classified as clinically significant as defined above. In contrast, none of the patients in whom telemetry was not indicated had a clinically significant arrhythmia (P < 0.05) (Fig. 3).

## Discussion

Telemetry monitoring, when applied appropriately, is a valuable resource for patients hospitalized with a range of diagnoses. However, telemetry is a limited resource in many hospitals and lack of telemetry can delay admissions from the Emergency Department.

It is perceived that telemetry provides a higher level of care than what can be provided on a general ward, but there is little data to support this. This level of care is maintained by an increased cost of nursing, telemetry technicians, and equipment. Thus, unnecessary admissions to telemetry translate into unnecessary added cost. In addition, the effect of the reduced availability of telemetry monitoring may be substantial on the hospital. It may result in trickle-down effect that potentially delays appropriate patient flow into and out of the emergency room, operating room and Intensive Care Units. The economic and personnel costs associated with the overuse of telemetry are significant. Further, incidental arrhythmias unrelated to the presenting complaint and/or of no clinical importance may trigger unnecessary additional diagnostic studies or unnecessary treatment. Worse, telemetry artifact is at times difficult to differentiate from arrhythmia. Artifact interpreted incorrectly as an arrhythmia may prompt diagnostic tests and therapies including anti-arrhythmic medications, pacemakers, and implantable cardioverter-defibrillators [4]. Despite these concerns, telemetry is frequently used though no suitable indication exists as seen in our study, where 25% of the admissions to the telemetry units had no indication based on our institutional telemetry monitoring guidelines.

Similarly, in a study by Curry et al., nearly one-third of patients admitted to telemetry monitoring had no indication for continuous monitoring [5]. Although many institutions have adopted the American College of Cardiology (ACC) guidelines for telemetry, few clinical studies have systematically measured the value of telemetry monitoring guidelines [6-12].

In a study done by Estrada et al. [7], they looked at the patients admitted to non-intensive care telemetry unit and pointed out that the role of telemetry in guiding patient management may be overestimated by physicians, it detected significant arrhythmias that led to change in medication or urgent interventions in small fraction of patient. Only 7% (156/2240) of the patients had direct modification in management and was perceived as useful but did not alter management for 5.7% (127/2240). In this study the author used the ACC guidelines for in-hospital cardiac monitoring, where 61% of the patients were assigned to ACC class I (telemetry indicated in most), 38% class II (telemetry indicated in some), and 1% class III (telemetry not indicated). In this study approximately 39% of the patients may not have met the criteria to be on telemetry leading the results found by the author. In comparison to our study, where patients were predefined by the authors as “telemetry indicated group” verses “telemetry not indicated group” according to our institutional guidelines, telemetry yielded higher clinically significant event rate in the “telemetry indicated group”. The study done by Estrada looked at overall event rate in all three groups together. In another study done by Estrada et al. [8], events in the individual class groups were looked at, telemetry led to change in management in 3.4% of the class I patients, 12.7% of the class II, and 4% of the class III. The study pointed out that if patients with chest pain as the reason for admission were moved from class I to class II and patients with arrhythmias as the reason for admission were moved from class II to class I, more arrhythmias and more clinically significant arrhythmias occurred in class I patients. In our study patients admitted with the diagnosis of chest pain and low likelihood of ACS were included in the “telemetry not indicated group” and patients admitted with the diagnosis of arrhythmia were included in the “telemetry indicated group” impacted our results showing that yield of telemetry is high if guidelines are employed properly.

Our institution developed guidelines to define which patient require monitoring and this study is to validate those guidelines. The guidelines identify a group of patients who are at a very low risk of having a clinically significant arrhythmia. One hundred and forty-four (34%) patients in the “telemetry indicated” group compared to only 16 patients (11%) in the “telemetry not indicated” group had at least one arrhythmic event on telemetry. Moreover, 36% of arrhythmias in the “telemetry indicated” group were deemed clinically significant, resulting in a change in treatment. This is in contrast to the “telemetry not indicated” group, where none of the patients had a clinically significant arrhythmia.

### Limitations

A primary limitation of this study was its retrospective nature. In addition, the use of telemetry was left to the discretion of the treating physician(s). Thus, those in the “telemetry indicated” group may have had a higher percentage of arrhythmias that prompted a management change related to differences in the patient population.

### Conclusion

The results of this study support the effectiveness of telemetry guidelines. Patients not meeting criteria for cardiac monitoring are unlikely to have a clinically significant arrhythmia and can safely be managed without telemetry. Cardiac rhythm monitoring is an important diagnostic tool whose utility can be maximized with the use of clear guidelines. If the guidelines are employed correctly, the yield of monitoring is high.
